# Entrustable professional activities in post-licensure training in primary care pediatrics: Necessity, development and implementation of a competency-based post-graduate curriculum

**DOI:** 10.3205/zma001144

**Published:** 2017-11-15

**Authors:** Folkert Fehr, Christoph Weiß-Becker, Hera Becker, Thomas Opladen

**Affiliations:** 1 Gemeinschaftspraxis für Kinder- und Jugendmedizin, Sinsheim an der Elsenz, Germany; 2 Gemeinschaftspraxis für Kinder- und Jugendmedizin, Husum, Germany; 3 Universitätsklinikum Heidelberg, Klinik Kinderheilkunde I, Heidelberg, Germany

**Keywords:** Post-licensure training in primary care, Primary care, Networked post-graduate programs, Primary care pediatrics, Practice

## Abstract

There is an absence of broad-based and binding curricular requirements for structured competency-based post-graduate medical training in Germany, and thus no basis for comparing the competencies of physicians undergoing training in a medical specialty (*Ärzte im Weiterbildung*). In response, the German Society of Primary Care Pediatrics’ working group on post-graduate education (DGAAP) has identified realistic entrustable professional activities (EPAs) in primary care, defined their number, scope and content, selected competency domains, specified required knowledge and skills, and described appropriate assessment methods. These guidelines are referred to as *PaedCompenda* and can be accessed electronically by educators in pediatric medicine; the use and effectiveness of these guidelines are monitored by the German Association for Medical Education’s committee on post-graduate education (GMA). Teaching and training in pediatric medicine should take EPAs into consideration. To accomplish this, phases dedicated to primary care should be integrated into formal medical specialty training. Primary care pediatrics must enhance the sites where such training takes place into learning environments that prepare physicians trainees and turn the practicing specialists into mentoring educators.

## Zusammenfassung

Für eine strukturierte kompetenzbasierte Weiterbildung fehlt eine flächendeckende, verbindliche curriculare Vorgabe und damit die Grundlage der Vergleichbarkeit der Kompetenzen von Ärztinnen in Weiterbildung (ÄiW). Die Arbeitsgruppe Weiterbildung der Deutschen Gesellschaft für Ambulante Allgemeine Pädiatrie (DGAAP) hat deshalb nach dem Konzept der *Entrustable Professional Activities* reale anvertraubare professionelle Tätigkeiten (APT) der Grundversorgung identifiziert, Anzahl und Umfang entschieden, Titel und Inhalte definiert, Kompetenzdomänen ausgewählt, erforderliches Wissen und notwendige Fähigkeiten spezifiziert und Prüfungsmethoden beschrieben. Diese Handreichung steht für pädiatrische Weiterbilder unter dem Namen *PaedCompenda* elektronisch zur Verfügung und wird in Anwendung und Wirksamkeit vom GMA-Ausschuss Weiterbildung begleitet. Die Praxis der Weiterbildung in der Pädiatrie sollte APTs berücksichtigen. Dazu ist die Einbindung ambulanter Abschnitte in der Facharztweiterbildung wünschenswert. Die primärversorgende Pädiatrie muss dazu ihre Lernorte zu vorbereiteten Lernumgebungen für Ärztinnen in Weiterbildung und ihre Praxisinhaber zu Lehrern weiterentwickeln.

## Introduction

### Needs and challenges

Post-graduate medical training in Germany is currently governed by the rules and regulations of the German Medical Association (Bundesärztekammer), and post-graduate programs vary widely between different medical disciplines as well as between programs in the same discipline. In pediatric medicine, up to 24 months of practice are required; however the majority of physicians receiving specialized post-licensure pediatric training do so in the hospital setting, while half of the specialists in pediatric and adolescent medicine practice as primary care practitioners.

Most children in Germany are seen in the practices of primary care pediatricians. The reasons for their visits to primary care practices have changed significantly. Today, pediatricians spend 25% of their time providing care for new morbidities and another 30% on early screening and immunizations. The physician trainees spend the majority of their time in the hospital, where the spectrum of pediatric and adolescent medicine has changed from general pediatrics to neonatal and premature infant care and the highly specialized care of rare diseases. Physicians newly established in primary care pediatrics indicated in a survey that they do not feel sufficiently trained to handle the difficult cases presented to them [1].

The provision of pediatric and adolescent heathcare is complexly organized in Germany, with German Social Codes V, VII, VIII, IX, XI and XII having relevance for child healthcare [2].

Planning medical care within the scope of the government-mandated health insurance is subject to various regulations and steering mechanisms. Opportunities to set up a primary care medical practice that participates in the government-mandated program are regulated and controlled by need-based planning. Federal planning guidelines classify pediatricians as general specialists and organize them according to the geographical regions defined in 1990. Over the past decade and a half, the diseases seen in children and adolescents, and as a result their needs, have changed significantly. This has given rise to paradoxes. On the one hand, the central institute for government-mandated health care in Germany calculates that across Germany there are 590 practicing pediatricians in excess of an upper limit of 140%, while complaints from children, adolescents and young adults indicate that healthcare needs are going increasingly unmet. How can children, adolescents, and their families receive excellent care today and in the future?

#### Challenges

- Even in large cities such as Stuttgart, parents have difficulties finding a primary care pediatrician for the routine preventive screening for four-week-old infants (U3). Medical practices in rural areas are finding no successors.
- Young practitioners feel poorly prepared to provide primary care. The German Society of Primary Care Pediatrics (DGAAP) conducted its own studies in 2013 on 196 pediatricians and found that 44% of those new to practice did not feel prepared to provide preventive screening for infants. A total of 50% felt unprepared to address problems with school; over 66% were unconfident about providing long-term care for chronically ill children.
- The hospital setting and private practice are increasingly drifting apart. Economic pressure and a supposed surplus of hospital beds allocated to pediatric care lead to ongoing and ever higher degrees of specialization.
- If pediatric medicine does not place emphasis on its overall interests, its voice will be drowned out by those of the other medical specialties.

#### Options

The curricular content of post-graduate training can be learned particularly well in the setting where the required skills are frequently practiced with routine and expertise. The DGAAP, with the support of the Professional Association of German Pediatricians (BVKJ), has proactively encouraged and realized research projects and designed a post-graduate program that not only reflects, but also does justice to the significant changes recently seen in pediatrics.

Literature research and our own research have revealed that the concept of entrustable professional activities (EPAs) proposed by the Dutch education scientist Olle ten Cate can furnish a future-oriented model covering core pediatric content.

#### Consequences for post-graduate training in pediatrics

Post-licensure practical training could form a core component of future networks among post-graduate programs. The confidence of physician trainees and mentor physicians in their own abilities would be strengthened, as would professional cooperation. Hospitals could profit in that physician trainees would be able to attend to patients requiring routine primary pediatric care. How will physician trainees, physician mentors, children and their families, and society react to the introduction of competency-based post-licensure training into primary care and what will it accomplish?

#### The concept of EPAs: Entrustable professional activities

Entrustable professional activities are a bridge between curricular content and the results of professional work – in this case excellent healthcare for children, adolescents, young adults and their families. EPAs describe activities, not competencies [3]. They are often professionally defined, since multiple healthcare professionals and other occupational groups participate in most of the activities.

According to ten Cate, the entrustable professional activity is:

1. part of a critical professional task within a particular context;
2. requires an appropriate level of expertise, skill and professional conduct;
3. yields recognized work as a result;
4. is limited to qualified personnel;
5. can be done virtually independently of other tasks;
6. can be executed within a given timeframe;
7. can be observed and measured as a process and a result (done well or not done well);
8. reflects one or more general medical skill(s).

## Method

### Selection of EPAs

Based on the work of ten Cate, Jones and Berberat et al., the DGAAP began to systematically explore the concept of the EPA for use in pediatrics. It was determined that the competency-based training aligned very well with the needs of children, families, and their pediatricians.

How can pragmatic change [4] be brought about? Since 2013 the DGAAP has followed the position paper of the GMA committee on post-graduate education and established a post-graduate training segment that was created by:

1. selecting EPAs for the curriculum in primary care pediatrics
  -identifying authentic EPAs
  - deciding the number and scope of the EPAs
2. Describing the EPAs
  - assigning titles and content to the EPAs
  - selecting the competency domains
  - specifying the requisite knowledge and skills
  - describing the assessment methods
3. Schedule for learning and testing the EPAs
  - determining the EPAs and the grading criteria over the course of the post-graduate program
  - coordinating the schedule in detail

#### Describing the EPAs

Competency domains are in certain respects the language of EPAs. Depending on the definition, focus is placed on content and references are made to curricular objectives. The current paper draws on the CanMEDS framework, widely known in Europe, and the six overlapping abilities or roles of the physician or medical expert: communicator, collaborator, manager, health advocate, scholar, and professional.

To specify the requisite knowledge and skills, the DGAAP applied a multi-step Delphi process in which pediatricians who were undergoing or had already completed specialty training were surveyed online regarding post-graduate education. The survey used approximately 600 items from the beta version of the DAKJ logbook and the Swiss catalogue of learning objectives. A four-point Likert scale was used ranging from 1=not important to 4=very important. Participants were divided into five groups based on occupational situation – university instructors, chief physicians at pediatric hospitals not aligned with a university, pediatricians involved in primary or tertiary care, and physician trainees – whereby each was given survey collection options. Items that were not definitively weighted were extracted and sent out again for response. The competencies asked about were viewed as being core content if they had a mean value of 3.6-4.0. From September 30, 2013, to August 5, 2016, a total of 889 responses were received. Since there is no established method for statistically demonstrating consensus among experts, reliance was placed on the definition of the strictest limit values possible. Items with the highest level of agreement were defined as core content. All of the groups were weighted the same in that the simple mean for each group is incorporated into the overall mean for the particular item. Consensus on an item was viewed as reached if it attained a mean value of 3.6-4.0 in the overall scoring. The detailed results are being published in another paper. In general, it was surprising that the DGAAP translation of CanMEDS into German qualified as being able to draw a consensus in the very first round. Since the National Competency-based Catalogue of Learning Objectives for Undergraduate Medical Education (NKLM) also uses this concept, the junction between undergraduate and post-graduate education is well established. EPAs are characterized by the competency domains which are also matched with concrete learning objectives by the Delphi study.

Another important source for the pediatric EPAs is the DAKJ study [5], which shed light on the frequency of and time required for doctor’s visits in primary care pediatrics. Based on this, decisions were made regarding the granularity of the EPAs. The care most needed in primary care pediatrics should be the most routine to give.

#### Quality assurance

Important post-graduate learning content must be dealt with or seen regularly by those responsible for post-graduate training. Respectful individualized feedback regarding observed activities is the strongest known driver behind professional development. For this reason, observational assessments in the workplace are a central element of competency-based post-licensure training. Physician trainees in pediatrics provide care for real children and their families during actual doctor’s visits. There is no extrapolation based on a multiple-choice test to guess what kind of doctor someone is or what kind of care he or she gives to patients. The result of good training is expert medical care, meaning that the results count and feedback on performance is a must. This scrutiny must be done in a respectful manner since the observational assessment serves as a model for doctor-patient relationships. It is impossible to care for patients without specialized knowledge and skills. But in terms of the observational assessment it is much more important to articulate thoughts and attitudes, feelings and emotions so that physician trainees can develop their skills, acknowledge their limits and ask for appropriate help in an environment based on trust. To enable this, the sub-competencies are listed as part of the EPA and reflected upon in feedback conferences between physician trainee and mentor. The decision to entrust the physician trainee with an activity of a particular difficulty can only be informed in part by previous observations. It will be necessary at first for the mentor and the trainee to speak with each other and make decisions on any necessary adjustments. This is why the BVKJ has called out for its regional associations to appoint people responsible for post-graduate training in primary care. These persons attend regular workshops with video recordings of EPAs and the feedback conferences so that they are able to more confidently make decisions about entrusting specific professional activities and better able to help others in this process.

## Results

To foster this development, the DGAAP has presented the competency-based curriculum for practice: the “core competencies.” Alongside treating preventable courses of disease, the focus on the DGAAP’s EPA curriculum is prevention and long-term care of chronically ill children.

Based on the preliminary groundwork, 12 EPAs were identified and assigned to learning objectives, and assessment formats were also compiled. In accordance with the principles of competency-based education, the focus shifts from norm-based to standard-based education and the fixed schedule before the exam to a flexible one. A pediatrician is not someone who has spent five years enrolled at an educational institution, but rather someone who has mastered the skills essential to this medical discipline (see table 1 (Tab. 1)).

**Figure 1:**
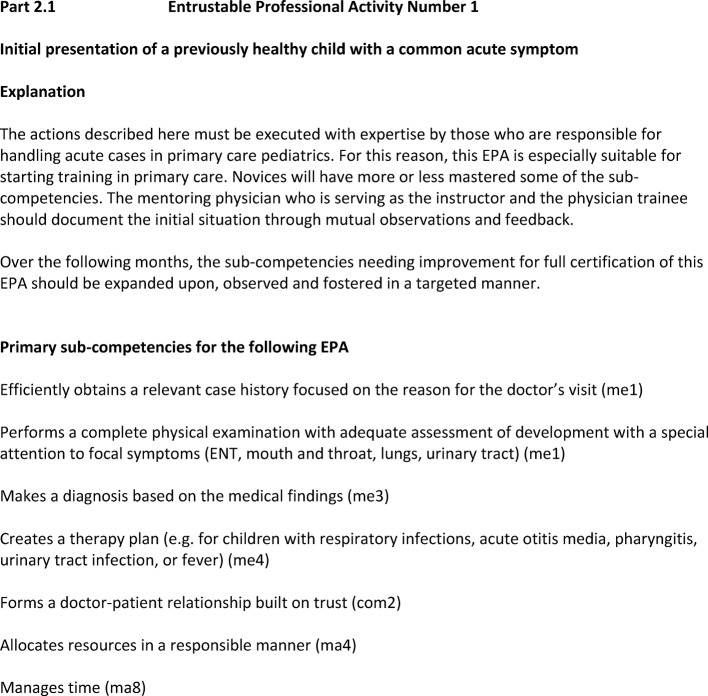
Description of the first EPA

Each EPA begins with a description (see figure 1 (Fig. 1)) in which the sub-competencies are clearly listed. A matrix ensures that all sub-competencies in the curriculum are covered and shows the frequency of the individual sub-competencies.

**Figure 2:**
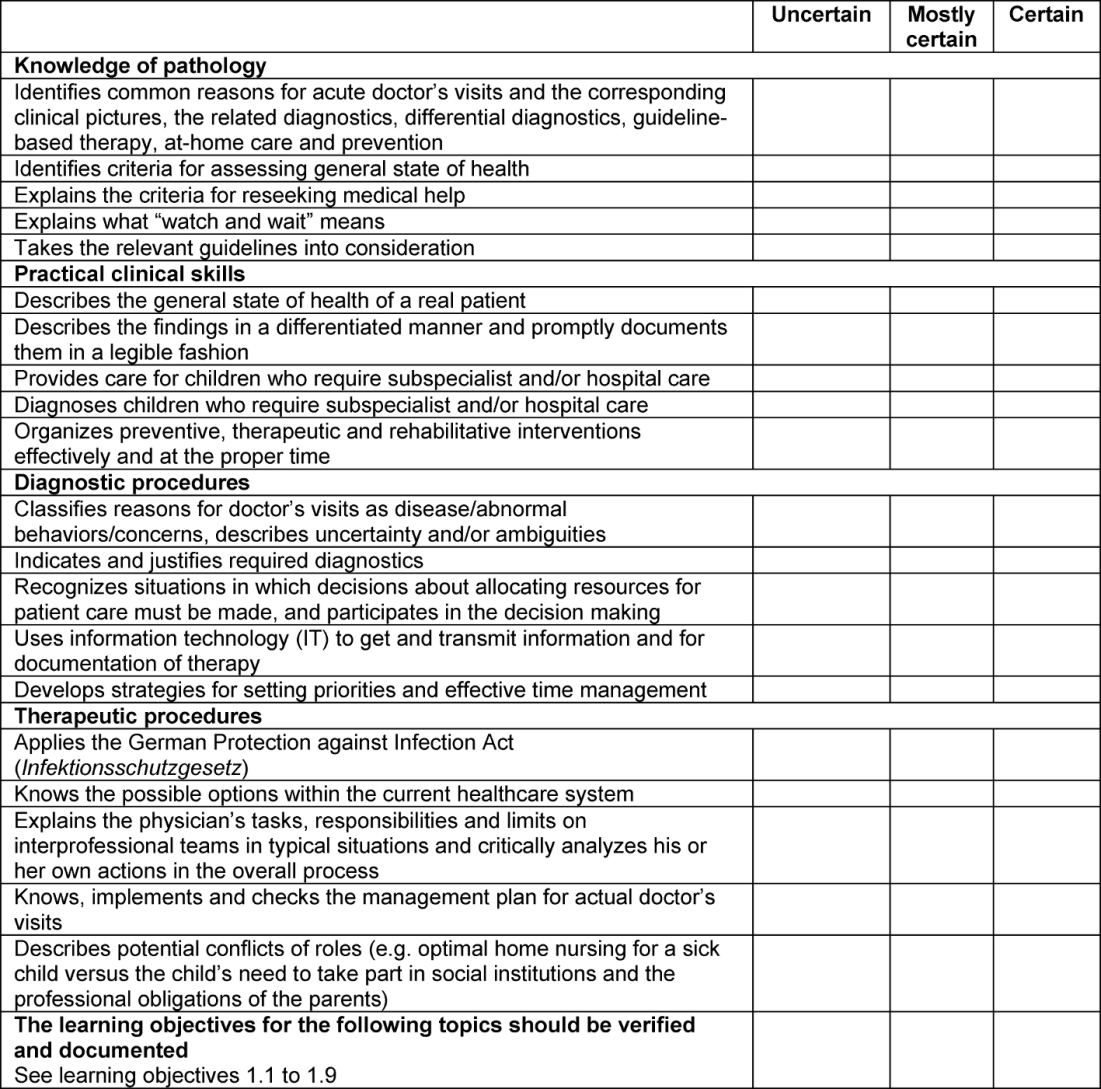
Learning objectives for “EPA 1.0 Initial presentation of a previously healthy child”

For each EPA there is a catalogue of learning objectives based on the Delphi process to identify core content in the post-graduate program for specialization in pediatrics (dissertation, Becker, H; currently being published). These lists of objectives primarily serve physician trainees and their physician mentors as they prepare for the observational assessments. In future it is planned to link these catalogues electronically with the logbooks used under the rules and regulations governing post-graduate training so that progress can be documented in real time (see figure 2 (Fig. 2)).

### Implementation of the post-graduate program in practice

To give expert medical care, physicians must continue their education. Agreement on the core content of such continuing education is vital as a means to identify authentic reasons for pediatric care and determine their nature and scope. Ascertaining the level of qualification – for instance the standard to be met by a medical specialist – is enabled by specific grading criteria.

Important aspects for putting competency-based education into practice include:

- Competency-based post-licensure training is evaluated based on criteria. This takes place on a daily basis while working in a medical practice and is described as an EPA. Drawing on the related list of learning objectives, each objective is worked on and achieved, and preparations are made for the observational assessments.
- Respectful feedback that provides motivation for personal and professional growth is important as the dominant tool of competency-based training. The observer, usually the mentoring pediatrician, is more a partner than a superior, and the relationship between mentor and trainee is not a hierarchical one, since both carry responsibility for the learning process. The learning experiences are assessed and reflected on jointly by trainee and mentor.

#### How can this be integrated into the daily operations of a normal pediatric medical practice?

Various forms of learning experiences are used more or less often over the course of the post-graduate training depending on expertise and progress. Activities can be observed during the different phases, the sequence of which depends on the prior knowledge of a particular physician trainee. Regardless of training segment, focus is always placed on joint appraisal by the physician mentor and the physician trainee: Which activities can be demonstrated by the trainee to the mentor, which activities still need to be carried out under supervision, and as of when can they be performed independently with the option of receiving feedback? And when can an activity be entrusted fully to the physician trainee?

##### 1. Preparatory phase

Physician trainee and mentor get to know each other. The EPAs are discussed, and the lists of learning objectives are initially used by both to establish the trainee’s post-graduate training status in primary care pediatrics: What activities have already been carried out independently? What has already been learned, seen, experienced? What activities are new? When reviewing the EPAs and the list of learning objectives, the trainee and mentor can gain an overview regarding the trainee’s experience and skills and the content of the post-graduate training in primary care pediatrics before beginning any practical work. Together they can plan and structure the course of the training. During the initial conferences and the introductory phase, trainer and mentor can form impressions of each other. It is clear from the start which activities the trainee has already carried out independently and if his or her level of confidence is strong enough to justify the assignment of personal responsibility for specific activities, or if detailed instruction and guidance are still needed.

##### 2. Introductory phase

This phase is shaped by the trainee‘s transition from observing to carrying out activities independently. At this point, the individual EPAs can be performed by the trainee in the presence of the mentoring physician, later independently with the option of asking questions and requesting assistance in making evaluations. The lists of learning objectives serve to ensure that important aspects of a specific activity are adequately addressed.

In the time that follows the trainee can assist the pediatrician by independently seeing patients who present for the very reasons described by the EPAs, while at the same time having the opportunity to work through and expand on other learning objectives by consulting with the pediatrician if there is any lack of certainty. As the scope of activities expands, the independent work of the trainee increasingly broadens. “EPA 1” – the acute doctor’s visit – lends itself to this phase. In the beginning these visits represent a great amount of time and effort for the pediatrician, but resources quickly free up in the practice once the physician trainee is able to assume responsibility for them.

##### 3. Consolidation phase

Step by step other activities can be assumed by the trainee according to the same principle. Different forms of learning are used to accomplish this: direct observation by the mentoring pediatrician during an authentic doctor’s visit or the trainee’s inclusion of the mentor in a visit when there are specific issues, challenging situations, or a lack of certainty regarding the diagnosis. Brief consultations between appointments, follow-up conferences or discussions of selected cases at scheduled times, and discussion of rare clinical pictures or specific medical issues based on actual patients round out the interaction between the two physicians. Observations and feedback on certain activities can be done by the mentoring pediatrician or delegated to trained personnel at the practice. An overall picture of the trainee’s progress can be seen in the numerous learning experiences. The aim should be to thoroughly cover the learning objectives during this phase.

##### 4. Entrusting phase

If the learning objectives for an EPA have been attained and if both trainer and trainee agree that an activity can be entrusted to the trainee, it is possible to grant the privilege of independent practice if the observational assessment has been successfully passed. The focus of the observation is on the core competencies described for each EPA during the introduction. Ten minutes should be planned for the observation and an appropriate amount of time for a follow-up conference and feedback.

If all of the observational assessments for all EPAs have been successfully passed, then the post-graduate training is completed in so far as the physician trainee has attained the privilege of independent practice, meaning the “license” – in the sense of a driver’s permit – to practice independently in primary care pediatrics.

Even in the future the trainee will strive, as will the mentoring pediatrician, to improve these skills and carry them out with utmost expertise.

## Discussion

The introduction of competency-based post-licensure training into primary care pediatrics has been met with both enthusiasm and reservation. Advocates see a great potential for trust in a network of physician trainees, physician mentors, children and families, and society at large. Critics see substantial amounts of extra work, bureaucracy, and the movement of sought-after physician trainees from hospitals into primary care. Ultimately, only scientific investigation and evaluation can show how *PaedCompenda* is used in practice and what its effects are. It will be necessary to include the expertise of specialists in medical education from the very beginning. Familiarity with *PaedCompenda* should be fostered, as well as a sense of ownership, through participation in its development, while at the same time discussion should be promoted regarding the concrete practical work and making the heterogeneity of the methods visible. The impression should be avoided that the teaching philosophy presented here is set in stone, ready for use and can be transferred directly from the undergraduate setting to post-licensure training. For what is of particular interest to pediatricians, as medical specialists for prevention and advocates of children and their families, is what effect these changes in post-graduation training have on the results of such training and on the health of children and adolescents.

## Competing interests

The authors declare that they have no competing interests. 

## Figures and Tables

**Table 1:**
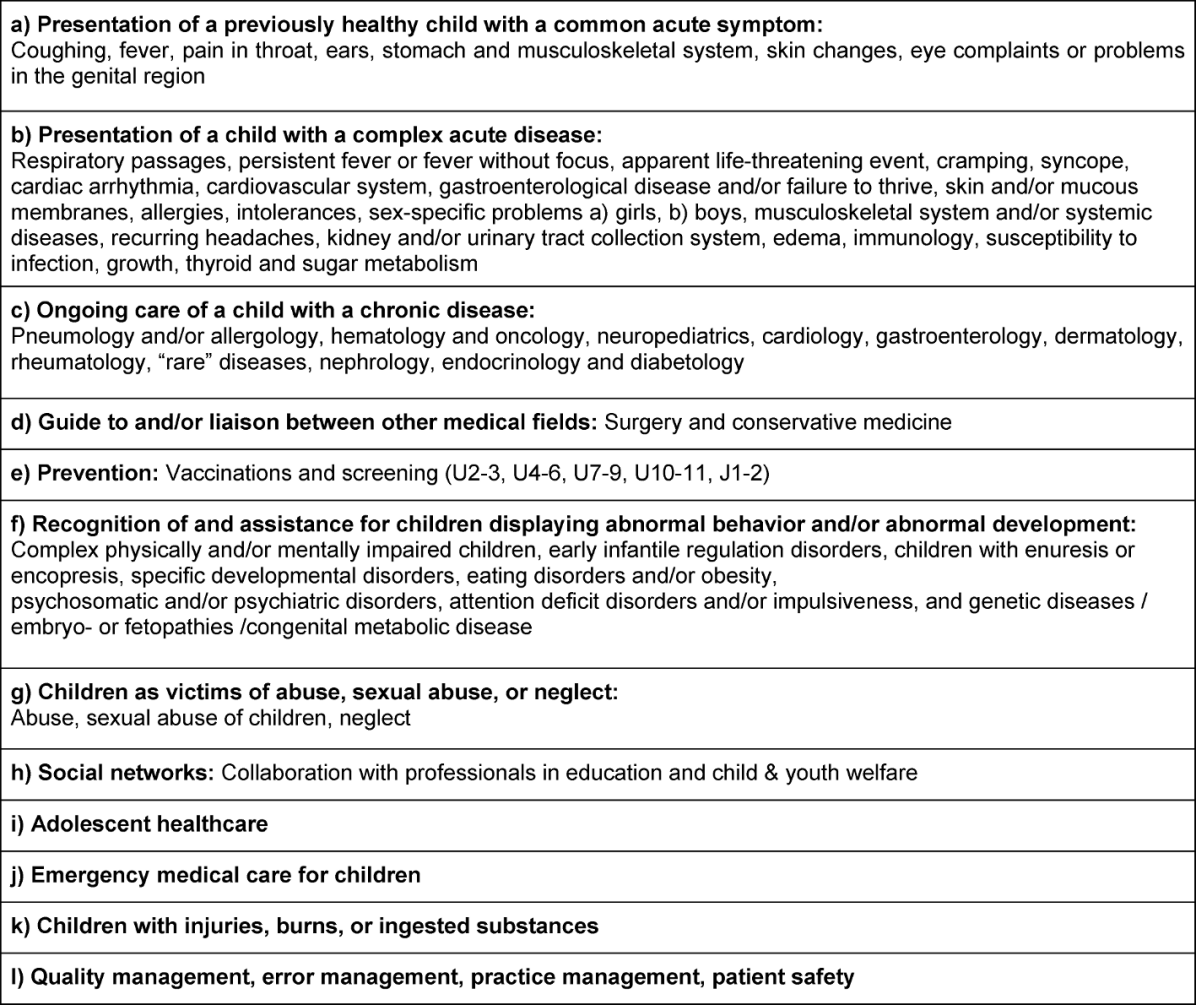
Overview of the DGAAP entrustable professional activities
